# The many weak instruments problem and Mendelian randomization

**DOI:** 10.1002/sim.6358

**Published:** 2014-11-10

**Authors:** Neil M Davies, Stephanie von Hinke Kessler Scholder, Helmut Farbmacher, Stephen Burgess, Frank Windmeijer, George Davey Smith

**Affiliations:** a Medical Research Council Integrative Epidemiology Unit, University of BristolBarley House, Oakfield Grove, Bristol, BS8 2BN, U.K.; b School of Social and Community Medicine, University of BristolBarley House, Oakfield Grove, Bristol, BS8 2BN, U.K.; c Department of Economics, University of Bristol8 Woodland Road, Bristol, BS8 1TN, U.K.; d Munich Center for the Economics of Aging, Max Planck Institute for Social Law and Social PolicyAmalienstr 33, 80799, Munich, Germany; e Department of Public Health and Primary Care, School of Clinical Medicine, University of CambridgeCambridge, CB1 8RN, U.K.

**Keywords:** Mendelian randomization, many weak instruments, continuously updating estimator, allele scores, height, ALSPAC

## Abstract

Instrumental variable estimates of causal effects can be biased when using many instruments that are only weakly associated with the exposure. We describe several techniques to reduce this bias and estimate corrected standard errors. We present our findings using a simulation study and an empirical application. For the latter, we estimate the effect of height on lung function, using genetic variants as instruments for height. Our simulation study demonstrates that, using many weak individual variants, two-stage least squares (2SLS) is biased, whereas the limited information maximum likelihood (LIML) and the continuously updating estimator (CUE) are unbiased and have accurate rejection frequencies when standard errors are corrected for the presence of many weak instruments. Our illustrative empirical example uses data on 3631 children from England. We used 180 genetic variants as instruments and compared conventional ordinary least squares estimates with results for the 2SLS, LIML, and CUE instrumental variable estimators using the individual height variants. We further compare these with instrumental variable estimates using an unweighted or weighted allele score as single instruments. In conclusion, the allele scores and CUE gave consistent estimates of the causal effect. In our empirical example, estimates using the allele score were more efficient. CUE with corrected standard errors, however, provides a useful additional statistical tool in applications with many weak instruments. The CUE may be preferred over an allele score if the population weights for the allele score are unknown or when the causal effects of multiple risk factors are estimated jointly. © 2014 The Authors. *Statistics in Medicine* published by John Wiley & Sons Ltd.

## 1. Introduction

Mendelian randomization uses genetic variants as instrumental variables to investigate the effects of modifiable risk factors for disease such as weight, blood pressure, cholesterol, alcohol, and tobacco consumption on different outcomes of interest [1–9]. An instrument is a variable external to the model of interest that is robustly associated with the modifiable risk factor, but is not associated with the outcome variable, other than through its effect on the risk factor. One challenge in using genetic instrumental variables is that many genetic variants are only modestly associated with the risk factor of interest, which limits the power and precision of a study. One approach commonly used in econometric studies to increase the power and precision is to include multiple instruments for the exposure of interest. However, studies using multiple instruments that are only modestly associated with the risk factor of interest can suffer from many weak instruments bias [10].

A weak instrumental variable is one that explains only a small proportion of the variation in the exposure. Studies using a single weak instrument will have low power to reject the null hypothesis. In addition, any minor violation in the ‘exclusion restriction’ (i.e. the assumption that the instrument is unrelated to any confounders and does not directly affect the outcome of interest) can cause large biases [11].

Studies using many instruments that are each only weakly associated with the exposure can suffer from a further distinct bias: a many weak instruments bias. This implies that, even if a set of multiple instruments is valid, (i.e. they are not associated with confounding factors, have no direct effect on the outcome, and are at least weakly associated with the exposure) the two-stage least squares estimator can still be biased towards the conventional regression estimate [10,12]. This is a concern in applications using multiple genetic variants as instruments, because each variant is typically weakly associated with the exposure of interest [13–16]. We demonstrate that, in agreement with previous theoretical and simulation results [17–19], weak instrument robust methods such as combining the variants into a single allele score, using the limited information maximum likelihood (LIML) estimator, or the continuously updating estimator (CUE) do not suffer from the many weak instruments bias. Whilst there are a number of techniques for using multiple variants, such as meta-analysis and structural mean models [20,21], the aim of this paper is to illustrate the use of CUE and LIML compared with 2SLS and allele scores. In addition, we show that the usual asymptotic standard errors for LIML and CUE are downward biased with many weak instruments. We therefore calculate corrected standard errors [18,22] and show that these can be used for correct inference. Finally, we illustrate these issues in the context of an empirical application, evaluating the effect of height on lung function.

The original example of the many weak instruments problem occurred in the work of Angrist and Krueger [23] who investigated the effects of length of schooling on wages and used quarter of birth as an instrument for time spent in school. In their basic specification, they used three variables indicating quarter of birth as instruments for length of schooling. However, these instruments explained relatively little of the variation in their exposure (partial *R*^2^=0.00012). In order to increase the precision of their estimates, the authors included the interaction of length of schooling and year of birth, which resulted in 180 instruments. This increased the partial *R*^2^ to 0.00043 and reduced the standard errors on the effect of schooling by over half. However, Bound *et al*. [10] and Hansen *et al*. [17] showed that, whilst the results were more precise, the point estimates from the instrumental variable regression were biased towards the ordinary least squares (OLS) estimates.

## 2. Methodology

In this section, we describe the different instrumental variables estimators applied here using the generalized method of moments (GMM) framework. OLS, two-stage least squares, and the CUE are all special cases of GMM. Consider a structural model with one continuous outcome, *l* continuous exposures, and *k* instrumental variables


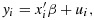
(1)

for individual observations *i* = 1,…,*n*, with *n* as the sample size, and where outcome *y*_*i*_ is a scalar, *x*_*i*_ is a *l* × 1 vector of the exposures, and *u*_*i*_ is a scalar indicating the error term. *β* is a *l* × 1 vector of parameters. Unobserved confounding implies that *u*_*i*_ is correlated with the exposure *x*_*i*_ and hence the OLS estimator is inconsistent. An estimator is inconsistent if it is a biased estimator of the causal effect even in large samples, whereas an estimator is consistent if it is an unbiased estimator of the causal effect in large samples: in other words, it is asymptotically unbiased. The *J* × 1 vector of instruments *z*_*i*_, with 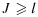, satisfy the population moment conditions


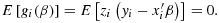
(2)

This results in one equation (moment condition) for each instrument, and the objective of the estimator is to find the value of *β* that minimizes a criterion function *Q*(*β*). The general form of a GMM criterion function is given by


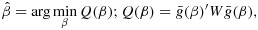


where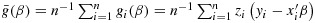 is the sample analog of the population moment conditions (2) and where *W* is a weight matrix giving potentially differential weight to each moment condition, which affects the efficiency of the GMM estimator. This weight matrix affects the properties of the estimator only if the model is overidentified; that is, when there are more instruments than exposures; *J* > *l*. Note that the OLS estimator is equivalent to a GMM estimator with *z*_*i*_ set equal to *x*_*i*_

### 2.1. Two-stage least squares

A widely used weighting matrix is 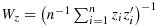, which, combined with the moment conditions earlier (2), results in the two-stage least squares estimator


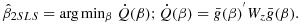
(3)

Setting the first derivative of 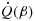 to zero and rearranging terms give the well-known formula for the two-stage least squares estimator


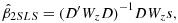
(4)

where 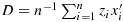 and 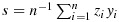. The two-stage least squares estimator is efficient, given moment conditions (2), when the variance of *u*_*i*_ is homoskedastic; that is, 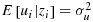. When the conditional variance of *u*_*i*_ is heteroskedastic, that is, a function of the instruments *E*[*u*_*i*_|*z*_*i*_] = *σ*^2^(*z*_*i*_), two-stage least squares is no longer efficient, but the two-step GMM estimator, as described later, is.

### 2.2. Two-step generalized method of moments (GMM)

Hansen described the two-step GMM estimator [24]. The two-step estimator uses a preliminary consistent estimate of *β*, denoted 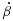 (e.g. 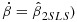, to compute the efficient weight matrix


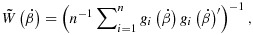


where 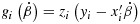. In the second step, the weight matrix is substituted into the objective function to obtain the two-step GMM estimator 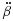



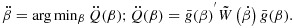
(5)

### 2.3. The continuously updating estimator (CUE)

The CUE proposed by Hansen *et al.* simultaneously minimizes over *β* in the moment conditions and the weighting matrix [25]. The CUE 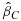 is defined as


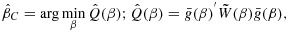
(6)

where 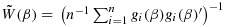. Note that the weight matrix now depends on *β*. Imposing conditional homoskedasticity, the CUE objective function simplifies to


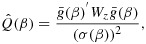
(7)

where 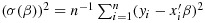. The estimator that minimizes this function is the same as the LIML estimator, and therefore, the CUE is identical to LIML when imposing conditional homoskedasticity. CUE has the same limiting distribution as the two-step GMM estimator under standard asymptotics with a fixed number of instruments and is hence efficient under conditional heteroskedasticity.

### 2.4. The bias with many weak instruments

Theoretical and simulation studies such as Newey and Windmeijer and Burgess and Thompson have demonstrated that LIML and CUE are less biased than two-stage least squares (2SLS) when using many weak instruments [17–19]. Under conventional, strong instrument, asymptotics, the bias of the 2SLS estimator when there are more than three instruments can be approximated by [10,26–28]


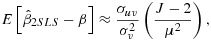
(8)

where the concentration parameter, 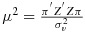, is the amount of the variation in the exposure that is jointly explained by the instruments, 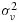 is the variance of the error term in the first stage equation for *x*, 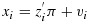, and *σ*_*uv*_ is the covariance of the error terms *u*_*i*_ and *v*_*i*_. In the sample, the concentration parameter evaluated at the OLS estimates for *π* and 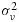 and divided by *J* is equal to the *F*-statistic for testing the null hypothesis that *π* = 0. Thus, the 2SLS bias shown in Equation (8) is inversely proportional to the concentration parameter and proportional to the number of instruments and covariance term. If a researcher adds an extra instrument, which increases *J*, the bias will only fall if it explains a sufficient additional amount of the variance in the exposure (i.e. it decreases the 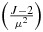 term). If the additional instrument only explains a small proportion of the variation in the exposure, the 2SLS bias will increase. This means that, when using 2SLS, researchers need to consider not only the strength of the association between each instrument and the exposure but also the number of instruments included.

The analogous bias approximation for LIML is given by [27,28]


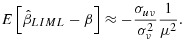
(9)

The bias of LIML is inversely proportional to the concentration parameter and negatively proportional to the covariance term but does not depend on the number of instruments. This latter feature is also valid for the CUE [29,30]. In other words, additional instruments can be added when using LIML/CUE as long as they increase the concentration parameter.

Limited information maximum likelihood and CUE are consistent and asymptotically normally distributed under many weak instrument asymptotics, where the number of instruments is allowed to grow with the number of observations [18,31]. 2SLS and two-step GMM are inconsistent estimators of the causal effect under these asymptotics. For both LIML and CUE, the usual asymptotic standard errors are too small when there are many weak instruments. Bekker [31] and Newey and Windmeijer [18] provide corrected standard errors for the LIML estimator and CUE respectively resulting in reliable inference when there are many weak instruments. We will base our inference later on these corrected standard errors. See Baum *et al.*, for a more detailed review [32]. Hausman *et al.*, [33] further show that LIML can be biased with many weak instruments when the errors *u*_*i*_ are conditionally heteroskedastic. As CUE remains efficient under these conditions, we argue that CUE is preferable to LIML for most applications.

## 3. Simulation study

We use a simulation study to illustrate the many weak instrument bias and the performance of the corrected standard errors for the LIML and CUE in Mendelian randomization. The data generating process has been described in detail previously [19]. In short, there is a single continuous exposure *x*, a continuous outcome *y*, and *J* instruments {*z*_*j*_}={*z*_1_,…,*z*_*J*_} that are discrete with values *k*={0,1,2}, mimicking the allele frequencies of genetic markers. We simulated data for three examples using different numbers of genetic variants as instrumental variables. Specifically, we set *J*= 9, 25, and 100 variants.

The data are generated from


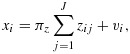
(10)


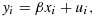
(11)


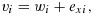



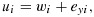



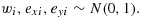
(12)

We set *π*_*z*_=0.1 for 9 variants, *π*_*z*_=0.06 for 25 variants, and *π*_*z*_=0.03 for 100 variants with a minor allele frequency of 0.3 for each variant. The exposure *x*_*i*_ has no causal effect on *y*_*i*_ (i.e. *β* = 0). There is, however, a positive correlation between *u*_*i*_ and *v*_*i*_ because of the presence of the unobserved confounder *w*_*i*_ in the models for both the risk factor and the outcome. We used this data generating process to simulate 10,000 datasets with 3000 observations each and report median bias and rejection proportions of Wald tests for the null hypothesis H_0_: *β* = 0. To avoid local optima, we estimated the CUE by taking the minimum of 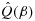 obtained from five different starting values. Specifically, we used {−2,−1,0,1,2} times the two-step GMM estimates as starting values.

Using the bias shown in Equations (8) and (9), we can estimate the theoretical bias. ‡ As the instruments are independently and identically distributed with variance


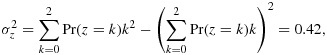
(13)
and 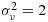, the concentration parameter simplifies to


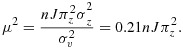
(14)

According to Stock *et al.*, [34], the expectation of the *F*-statistic minus one approximately equals the concentration parameter divided by the number of instruments or *E*(*F*) ≅ *μ*^2^/*J* + 1. Thus, solely based on the parameters of the data generating process, we know the concentration parameter, the approximate expectation of the *F*-statistic, and the approximate bias of 2SLS and LIML estimators. We report these theoretical results together with the corresponding results from the simulation study in Table I. Furthermore, we report the rejection frequencies of the Wald tests for 2SLS, LIML, CUE, and the allele score instrumental variable estimators, calculated on the basis of the asymptotic and the corrected standard errors. The 2SLS estimator, LIML estimator, and CUE use the full set of instruments, not imposing any restrictions on the parameter vector *π*. The allele score is constructed as the unweighted sum of the instruments, which in this case is the sum of the risk alleles (10).

**Table I:** Simulation results comparing the properties of two-stage least squares, LIML, CUE, and allele score instrumental variable estimators when using 9, 25, and 100 variants as instrumental variables.

	Theoretical results		Empirical results		
*k*	Estimator	Bias	Median	IQR	RF
9	2SLS	0.057	0.065	0.158	0.101
	LIML	−0.009	0.002	0.186	0.057
	Corrected LIML				0.047
	CUE		0.002	0.187	0.079
	Corrected CUE				0.045
	Allele score IV		0.001	0.178	0.047
25	2SLS	0.147	0.150	0.139	0.321
	LIML	−0.009	0.001	0.204	0.079
	Corrected LIML				0.049
	CUE		0.002	0.207	0.159
	Corrected CUE				0.046
	Allele score IV		0.000	0.181	0.044
100	2SLS	0.318	0.318	0.095	0.988
	LIML	−0.009	0.002	0.277	0.173
	Corrected LIML				0.046
	CUE		0.001	0.297	0.481
	Corrected CUE				0.045
	Allele score IV		0.000	0.176	0.042

Note that *μ*^2^=56.70 in all designs. *E*[*F*]≈7.30,3.27,1.57 for *k* = 9,25,100, respectively. The means of the empirical *F*-statistics are 7.40, 3.32, and 1.58. The rejection frequency is the proportion of replications for which the true parameter (*β* = 0) lies outside the 95% confidence intervals. Median, median estimate, *β* = 0; *n* = 3000; 10,000 replications. 2SLS rejection frequencies assume a homoskedastic error term. Rejection frequencies for H_0_:*β* = 0 using Wald tests.

LIML, limited information maximum likelihood; CUE, continuously updating estimator; 2SLS, two-stage least square; IQR, interquartile range; RF, rejection frequency.

Table I presents medians and interquartile ranges (IQR) of the estimators. As the LIML estimator and CUE have occasionally very large outliers, this affects the mean and variance of these estimators, but not the median and IQR. The results show that, as expected, 2SLS suffers from the many weak instruments bias, whereas LIML and CUE are approximately unbiased in all simulations. The allele score instrumental variable estimator is also unbiased. Keeping the number of instruments fixed, all estimators have the same limiting distribution when the sample size increases. However, in the generated samples, only the allele score instrumental variable estimator displays a finite sample behavior close to the asymptotic distribution. Both LIML and CUE show larger finite sample dispersions than the allele score instrumental variable estimator as the number of instruments increases. The usual asymptotic standard error for LIML underestimates its true variability in this design, in line with previous work [18,19]. We also find this for the uncorrected asymptotic standard errors of the CUE. In contrast, the Wald tests based on the Bekker standard errors for LIML and the Newey–Windmeijer standard errors for the CUE show rejection frequencies close to the nominal size of 0.05 in all three simulations. Thus, the corrected standard errors make valid inference possible in situations with many weak instruments.

## 4. Empirical example: height and lung function

We illustrate the many weak instrument bias and its different estimators in an empirical application, investigating the relationship between height and lung function. We chose this example, because we know that there is a mechanical relationship between the two variables, with lung volume increasing with height. Therefore, we can use this example to illustrate how height variants can estimate the causal effect of height independent of a confounding factor – gender. Gender confounds the raw association of lung function and height because boys are both taller and have higher lung function. We used data from Avon Longitudinal Study of Parents and Children (ALSPAC), a cohort study of 15,247 pregnancies delivered between 1 April 1991 and 31 December 1992 in the South West of England, which has been described in detail elsewhere [35,36]. Our sample includes all 3631 individuals with genome-wide data, information on height and lung spirometry (forced vital capacity) when aged between 14 and 17 years. Ethical approval for the study was obtained from the ALSPAC Ethics and Law Committee and the Local Research Ethics Committees. The study website contains details of all the data that are available through a fully searchable data dictionary [37].

### 4.1. Outcome – lung function

Lung function was measured using forced vital capacity derived from a lung spirometry test. This measure has been described in detail elsewhere [38,39]. We standardized this measure to mean zero and standard deviation one.

### 4.2. Exposure – height

Individuals' height was measured in millimeters using the Harpenden stadiometer, when the individuals were aged 15 and a half [40]. We standardized height to mean zero and standard deviation one.

### 4.3. Covariates

We derived the following covariates: age at measurement (in months); birth weight; number of younger and older siblings; log weekly household income adjusted for inflation, household size, and composition; parents' education; and social class and employment status. Individuals whose fathers were in the military were coded as social class 3. The derivation of these covariates has been described elsewhere [41].

### 4.4. Instruments

**Figure 1:**
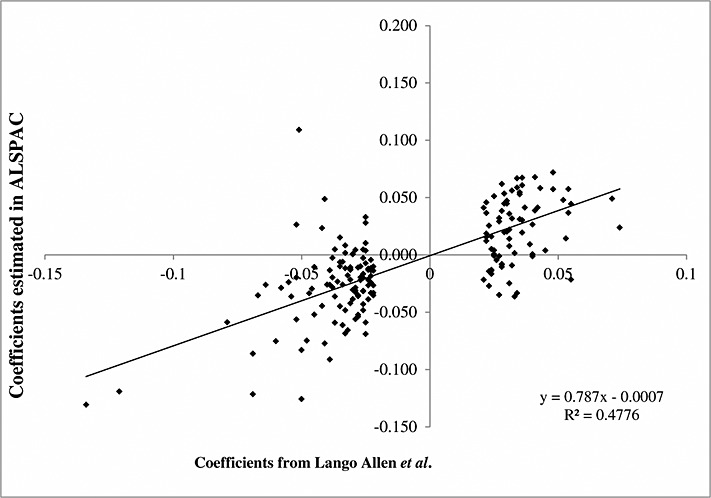
Plot of the association of 180 genetic variants and height in ALSPAC and Lango *et al.*, [42].

In a genome-wide study of 183,727 people, Lango *et al.* reported 180 independent genetic variants associated with height [42]. Because the ALSPAC cohort was not used in Lango *et al.,* our results will not suffer from bias due to selecting variants based on their observed association within our data [19]. We use the same 180 variants listed in Table S1, which shows their effect on height as reported by Lango *et al.*, as well as in our ALSPAC sample. In addition, Figure 1 plots the association of the coefficients estimated in our data and the coefficients reported by Lango *et al.* The protocols for cleaning the genome-wide data have been described in detail elsewhere [43,44].

We used the 180 height variants as 180 individual instruments. Each variant was coded 0, 1, or 2 depending on the combination of height-increasing alleles the individual had. To further explore alternative definitions, we also consider two additional versions of the instrument. First, a simple count of the number of height-increasing alleles


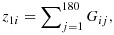
(15)

where *G*_*ij*_ indicates the *j*th genetic variant. Second, a weighted allele score, where the weights, 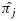, denote the strength of the association of the alleles of variant *j* and height, as reported by Lango *et al.*


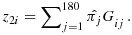
(16)

To simplify the interpretation of the results, we normalized the unweighted and weighted allele scores to have mean zero and standard deviation one.

### 4.5. Descriptive statistics

We tested whether the height, the height variants, and the allele scores were associated with the covariates using robust linear regression. Furthermore, we used linear regression to estimate the associations of 153 pairwise correlations of the 18 nongenetic covariates, and the 3240 pairwise associations of the 180 genetic variants and the 18 nongenetic covariates. We compared the number of significant associations (at *α* = 0.05, 0.01, and 0.001) that occurred to the number that would be expected if all the variables were uncorrelated. Finally, we tested the strength of the association of the instruments and height using linear regression and report the *F*-statistics for this association. We further report *R*-squared values as measures of the proportion of the variance in height explained by each definition of the instruments.

We compared the different instrumental variable methods for estimating the effect of the height on lung function with conventional unadjusted OLS regression of lung function on height and the same association adjusted for all the covariates mentioned earlier. We used the user-written ice command in Stata to impute missing values of the covariates for the adjusted OLS analysis [45,46]. We do not adjust any of the instrumental variable results for covariates. We report five versions of instrumental variable estimation results using all individual variants as instruments: (i) 2SLS, (ii) LIML, (iii) LIML with corrected standard errors, (iv) CUE, and (v) CUE with corrected standard errors. We further present results for (vi) 2SLS using an unweighted allele score as a single instrument and (vii) 2SLS using a weighted allele score as a single instrument.

For each estimation method, we report – where possible – the weak identification *F*-statistic, Hausman endogeneity test, and the Sargan or Hansen *J*-test of overidentifying restrictions [24,47–51]. The Hausman test evaluates whether there is any evidence that the instrumental variable estimate differs from the OLS estimate. The Sargan and Hansen *J*-tests of overidentification evaluate whether there is any evidence of difference between the instrumental variable results based on each variant. The Sargan test assumes conditional homoskedasticity, whereas the Hansen *J*-test is robust to conditional heteroskedasticity [24,52]. Hence, the Sargan test can be more powerful but misleading when conditional heteroskedasticity is present. We ran all analyses using Stata 13.0. The instrumental variable results were estimated with the user-written commands ivreg2 [53,54]. We report the corrected standard errors for both CUE and LIML using the user-written commands nwind and bekker [18,22,31].

To illustrate how the many weak instrument bias affects the results in the analysis that includes the individual variants, we sorted the height variants in order of effect size and repeated the analysis adding one variant at a time, starting with the variant with the biggest effect and ending with the weakest variant. We report the estimates and standard errors for each of the regressions using 2SLS and the CUE and also report *F*-statistics for the hypothesis that the concentration parameter equals zero (*μ*^2^=0).

## 5. Results

After cleaning and imputation, there were 8365 individuals with genome-wide data. We excluded data from 4734 individuals, who had missing measures of either height or lung function at age 15 years, which resulted in a final analysis sample of 3631. The characteristics of the individuals are described in Table II. Table III shows that the individuals' actual height at age 15 years was strongly associated with 9 of the 17 covariates (*p* < 0.05). Taller individuals were more likely to be from a higher-income household, have more educated parents and grandparents, have been breast-fed, and have an older mother. In contrast, there was little evidence of associations between the allele scores and the covariates (Table IV). Assuming no associations between the nongenetic covariates (153 possible pairwise correlations), we would expect to detect eight associations at the 5% significance level, two at the 1% significance level, and less than one at the 0.01% significance level. We detected 82 (54%), 70 (46%), and 54 (35%) associations at the 5%, 1%, and 0.01% significance level, respectively. For the 3240 genetic pairwise associations with the nongenetic covariates, we would expect to detect 162, 32, and less than one association at the 5%, 1%, and 0.01% significance level, respectively. We detected 166, 35, and no associations, respectively. This is consistent with the previous studies [8,55].

**Table II:** Baseline characteristics of included ALSPAC participants.

	Mean	Standard deviation	*N*
Height at age 15 years (in cm)	169.35	8.42	3631
Male	0.48	0.50	3631
Age in months at Teen Focus 3 Clinic	185.31	3.58	3631
Birth weight (g)	3444	525	3435
No. of older siblings in household	1.02	0.99	3319
No. of younger siblings in household	0.92	0.95	3319
Ln(income)	5.74	0.46	3141
Mother's education	3.39	1.18	3391
Father's education	3.39	1.37	3320
Mothers' mother's education	2.35	1.33	2627
Mothers' father's education	2.57	1.46	2471
Child not ever raised by natural father	0.11	0.32	3314
Father's social class at birth	2.78	1.27	3146
Mother works part time	0.43	0.50	3113
Mother works full time	0.09	0.28	3113
Partner works full time	0.93	0.25	1608
Mother drank during pregnancy	0.57	0.50	3419
Mother smoked during pregnancy	0.15	0.36	3418
Ever breast-fed	0.86	0.34	3226
Mother's age	29.82	4.51	3495
Participant had tried tobacco at age 8 years	0.02	0.15	3133

**Table III:** Association of height at age 15 years with baseline characteristics.

	Actual height		
	*N*	Coef	*p*-value
Age in months at Teen Focus 3 Clinic	3631	0.14	0.02
Male	3631	0.29	<0.001
Older siblings in household	3321	0.00	0.98
Younger siblings in household	3321	0.02	0.30
Ln(income)	3141	0.02	0.03
Mother's education	3391	0.06	0.002
Father's education	3320	0.08	0.001
Mothers' mother's education	2627	0.06	0.02
Mothers' father's education	2471	0.06	0.05
Child not ever raised by natural father	3314	0.00	0.62
Father's social class at birth	3146	−0.04	0.07
Mother works part time	3113	0.00	0.72
Mother works full time	3113	0.01	0.07
Partner works full time	1608	0.00	0.57
Mother drank during pregnancy	3419	0.00	0.85
Mother smoked during pregnancy	3418	−0.01	0.08
Ever breast-fed	3226	0.02	<0.001
Mother's age	3495	0.27	<0.001
Participant had tried tobacco at age 8 years	3133	0.01	0.03

Coef, coefficient from a robust ordinary least squares regression of covariate on normalized height.

**Table IV:** Association of variants and allele scores with covariates.

	Unweighted allele score		Weighted allele score	
	Coef	*p*-value	Coef	*p*-value
	(1)	(2)	(3)	(4)
Male	0.01	0.41	0.01	0.33
Birth weight (g)	31.47	<0.001	34.43	<0.001
Older siblings in household	0.01	0.60	0.01	0.45
Younger siblings in household	−0.02	0.19	−0.02	0.25
Ln(income)	0.00	0.62	−0.01	0.37
Mother's education	0.00	0.94	0.00	0.99
Father's education	0.00	0.94	0.00	0.84
Mothers' mother's education	0.02	0.50	0.02	0.42
Mothers' father's education	0.02	0.50	0.03	0.29
Child not ever raised by natural father	0.00	0.51	0.01	0.36
Father's social class at birth	0.00	0.83	0.00	0.99
Mother works part time	0.01	0.15	0.01	0.24
Mother works full time	0.00	0.66	0.00	0.50
Partner works full time	−0.01	0.21	−0.01	0.08
Mother drank during pregnancy	−0.01	0.52	−0.01	0.25
Mother smoked during pregnancy	−0.01	0.35	−0.01	0.18
Ever breast-fed	0.00	0.58	0.00	0.71
Mother's age	−0.09	0.21	−0.09	0.23
Participant had tried tobacco at age 8 years	0.00	0.58	0.00	0.32

The associations between the individual genetic variants and height were similar to the weights reported by Lango *et al.* (*R*^2^=0.478) (Table S1 and Figure 1). Table V shows that there is a strong association between individuals' height and the allele scores. The *F*-statistics for the unweighted and weighted allele scores were 164 and 190, respectively, suggesting that the results based on these instruments are unlikely to suffer from weak instrument bias [12]. A one-standard-deviation increase in the unweighted allele score was associated with a 0.21 (95% confidence interval (CI): 0.18, 0.24) standard deviation increase in height at 15. A one-standard-deviation increase in the weighted allele score was associated with a 0.22 (95% CI: 0.19, 0.25) standard deviation increase in height at 15. The unweighted and weighted allele scores explained 4.3% and 4.9% of the variation in height, respectively.

**Table V:** Association of allele scores and normalized height at age 15 years (*N* = 3631).

	Unweighted score mean difference		Weighted score mean difference	
	(95% confidence interval)	*p*-value	(95% confidenceinterval)	*p*-value
Allele score	0.21 (0.18,0.24)	<0.001	0.22 (0.19,0.25)	<0.001
*R*^2^	0.043		0.049	
*F*-statistic	164		190	

**Figure 2:**
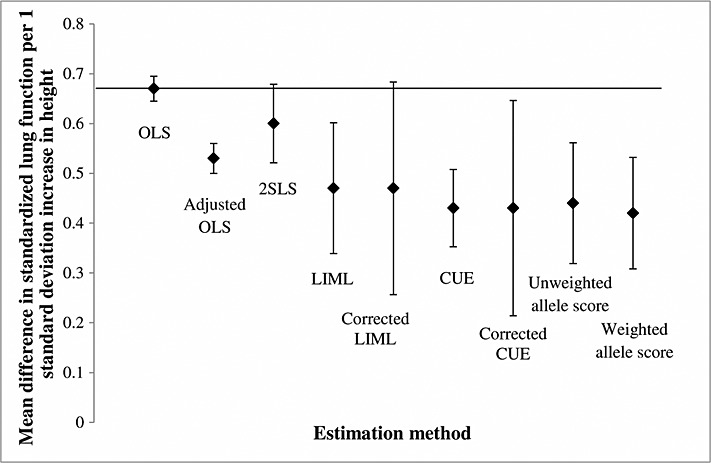
Estimated effect of standardized height on lung function. These results are presented in Table VI. OLS, ordinary least squares; 2SLS, two-stage least squares regression; LIML, limited information maximum likelihood; CUE, continuously updating estimator. The horizontal line indicates the OLS estimate. Lung function standardized to mean zero and standard deviation one.

Using conventional OLS regression, we found that a one-standard-deviation increase in height was associated with a 0.67 (95% CI: 0.65, 0.70) standard deviation increase in lung function (Table VI and Figure 2). After we adjusted for the observed covariates, this association was attenuated to 0.53 (95% CI: 0.50, 0.56). The 2SLS estimates using the individual alleles of all variants suggested that a one-standard-deviation increase in height caused a 0.60 (95% CI: 0.52,0.68) standard deviation increase in lung function (Table II and Figure 2). The 2SLS estimates are relatively close to the OLS estimates. The LIML estimate suggested that a one-standard-deviation increase in height caused a 0.47 (95% CI: 0.34, 0.60) standard deviation increase in lung function, similar to the CUE estimate. The corrected standard error for the CUE is virtually identical to the Bekker standard error for LIML. The Sargan/Hansen tests for the 2SLS, LIML, and CUE results suggested some evidence of differences between the estimates based on each variant.

**Table VI:** The relationship between height and lung function (*N* = 3631).

Method	Mean difference (95% confidence intervals)	Robust standard error	*p*-value	*F*-statistic	Sargan/Hansen *J*-test *p*-value	Hausman endogeneity tests	*p*-value
Ordinary least squares	0.67 (0.65,0.70)	0.013	<0.001				
Adjusted OLS	0.53 (0.50,0.56)	0.015	<0.001				
Two-stage least squares	0.60 (0.52,0.68)	0.040	<0.001	2.03	0.02	4.08	0.04
LIML	0.47 (0.34,0.60)	0.067	<0.001	2.03	0.01*	3.70	0.05
LIML corrected	0.47 (0.25,0.68)	0.109	<0.001				
CUE	0.43 (0.35,0.50)	0.039	<0.001	2.03	0.05		
CUE corrected	0.43 (0.21,0.64)	0.110	<0.001				
Unweighted allele score	0.44 (0.32,0.56)	0.062	<0.001	164.18		16.80	<0.001
Weighted allele score	0.42 (0.31,0.53)	0.057	<0.001	190.15		22.71	<0.001

Robust confidence intervals. Adjusted OLS adjusts for covariates described in Table II.

OLS, ordinary least squares; LIML, limited information maximum likelihood; CUE, continuously updating estimator.

* We used the Sargan test for LIML and the Hansen *J*-test for the other estimators. The Hausman test assumes homoskedasticity; therefore, we do not include it for CUE.

The instrumental variable results using an unweighted allele score were close to the LIML and CUE results, but the latter's uncorrected standard errors were 8.3% smaller and 36.2% smaller, respectively. The weighted allele score results were almost identical to the unweighted allele score, but the standard error was reduced by an additional of 7.9%. The standard errors of the weighted allele score estimates are 48% smaller than those from the corrected CUE or the corrected LIML analyses. As shown by the Hausman test, the allele score specifications found strong evidence of differences between the OLS and instrumental variable estimates.

**Figure 3:**
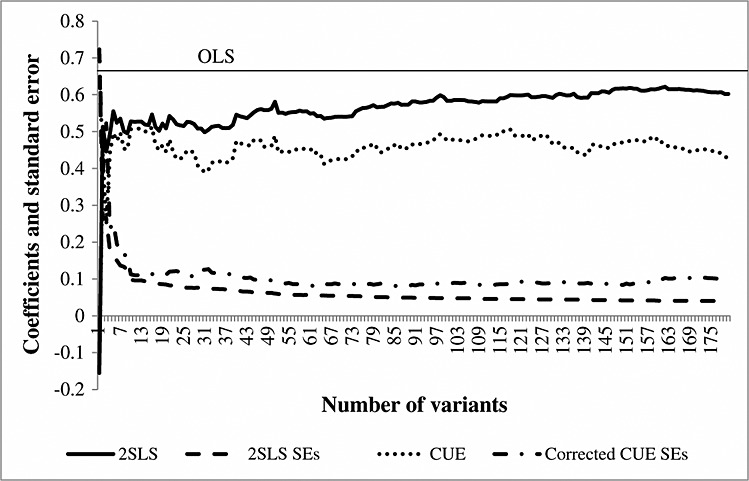
Coefficients and standard errors from two-stage least squares and continuously updating estimator by number of variants that are included as instruments. Variants included in order of association with height; thus, the analysis with one variant uses the strongest variant reported by Lango *et al*., and the analysis with two variants uses the two strongest variants and so on.

As we have shown earlier, the 2SLS estimate using all variants as instruments is relatively close to the OLS estimate. This is likely to be because 2SLS is biased towards OLS when using many weak instruments [10]. We illustrate the consequences of the many weak instrument bias in 2SLS further in Figure 3 and Figure S1. When we use fewer than 15 variants, the 2SLS and CUE estimates are similar. However, as we move along the *x*-axis and add more variants, the 2SLS estimates trend upwards, becoming more biased towards the OLS estimate, whilst the CUE remains relatively stable.

## 6. Discussion

When using many weak instruments in Mendelian randomization studies, researchers should consider the fact that the routinely used 2SLS estimator is biased. So far, two methods have been proposed in the epidemiology and econometric literature to account for this problem. First, constructing a weighted or unweighted allele score and using 2SLS with only one instrument. Second, using the full set of instruments together with an estimation method that is robust to using many weak instruments. In the latter case, only LIML has been discussed in the literature [13]. However, the conventional standard errors for LIML are incorrect with many weak instruments, leading to inaccurate inference for testing hypotheses. We have developed a Stata routine to calculate corrected standard errors for LIML to account for the many weak instruments problem and show that these adequately reflect the decrease in precision of the LIML estimator for these cases.

The LIML has recently been shown to be biased with many weak instruments when the errors are conditionally heteroskedastic [33]. The CUE is consistent under these conditions, and therefore, the CUE would be the estimator of choice in most empirical applications; for example, when estimating risk differences for binary outcomes. However, its standard errors also need to be adjusted when using many weak instruments. We have developed a Stata routine also for the CUE to adjust the standard errors and, again, show that these adequately reflect the decrease in precision when using many weak instruments.

Using a single allele score as instrument can be a useful and appropriate way to get around the many weak instruments problem. In contrast, the CUE will be useful in situations when there is no clear way to aggregate a set of instruments. For example, consider a researcher who is interested in the relative contributions of body mass index (BMI), fat mass and lean mass to asthma [56]. Whilst 32 genetic variants have been shown to associate with BMI, there have been no genome-wide association studies specifically for fat and lean body mass. Granell *et al.*, [56] used the same weighted allele score as an instrument for lean and fat mass as the one derived from a meta-analysis for the gene–BMI relationship. As the weights are unlikely to be the same for lean and fat mass, efficiency may be enhanced by using the CUE and the 32 BMI variants to accurately test whether BMI, lean or fat mass affects asthma.

The CUE may be particularly useful in cases where there are multiple risk factors of interest and many variants, as it may be difficult then to construct different allele scores for each of the risk factors. Indeed, in the example earlier, one could use the CUE to estimate the causal effects of lean and fat mass jointly. Outside the world of Mendelian randomization, recent pharmaco-epidemiological studies have found that including multiple physicians' previous prescriptions as instruments for their prescribing behavior increases the precision of estimates [57–59]. These results may be improved upon by using the CUE.

In this proof of principle study, we showed how to apply these methods using the illustrative example of the effect of height on lung function. We found that the height variants and allele scores were associated with height and that the genetic variants were no more associated with the observed covariates than would be expected by chance. Our results suggest that, compared with the causal effects identified by the genetic variants, the observational association may suffer from a slight upward bias. We demonstrated that the 2SLS estimates suffered from many weak instruments bias when we used all the individual variants. In contrast, the results using LIML, CUE, and the weighted or unweighted allele scores instrumental variable estimators were mutually in concordance, and all suggested a weaker underlying causal relationship between height and lung function. As with the simulated results, in the empirical example, the corrected standard errors for LIML were almost identical to the corrected CUE standard errors but considerably larger than those for the weighted allele score instrumental variable estimates. This is likely to reflect the relative efficiency of these approaches in many applied examples. Nevertheless, by holding the number of instruments constant, as the sample size increases, the difference in efficiency between these approaches will reduce.

In conclusion, when using many weak instruments, 2SLS estimates can be biased. We demonstrated that, under homoskedasticity, the allele score instrumental variable estimators, CUE, and LIML with corrected standard errors provide accurate inferences. If homoskedasticity holds, the LIML is likely to be slightly more efficient than the CUE. In small samples, both estimators are likely to be less efficient than the allele score. However, if there is conditional heteroskedasticity, LIML can be inconsistent when using many weak instruments and researchers should use the CUE instead. However, both methods are likely to be less efficient than using allele scores when there are many instruments and modest sample sizes. Using multiple variants as instruments can increase precision of the results over using single variants, but researchers must report corrected standard errors.

## Notes

‡ We calculate the bias for the 2SLS estimator from the improved formula as given by Bun and Windmeijer [26]. The results are very similar to those of 8.
