# Supplementary material for: The many weak instruments problem and Mendelian randomization
Source: Stat Med. 2014 Nov 10;34(3):454–68. doi: 10.1002/sim.6358 (PMC4305205; doi:10.1002/sim.6358)
Supplement: Supplementary file 1 [file sim0034-0454-sd1.docx]

eFigure 1: Coefficients and standard errors from two-stage least squares and continuously updating estimator by number of variants that are included as instruments (limited to up to 30 instruments).

Note: variants included in order of association with height, thus the analysis with one variant uses the strongest variant reported by Lango Allen et al. (2010), and the analysis with two variants uses the two strongest variants and so on.

eTable 1: Association of Normalized Height Aged 15 with Individual Height SNPs (N=3,631).

|  |  |  |  |  |  | Lango |  |  |
| --- | --- | --- | --- | --- | --- | --- | --- | --- |
|  |  |  |  |  |  | Allen |  |  |
|  |  |  |  | Effect | Other | et al. | ALSPAC |  |
| SNP | Chr | Position | Gene | allele | allele | beta | beta | Diff. |
| (1) | (2) | (3) | (4) | (5) | (6) | (7) | (8) | (9) |
| rs425277 | 1 | 2059032 | ***PRKCZ*** | T | C | 0.024 | 0.022 | 0.002 |
| rs2284746 | 1 | 17179262 | ***MFAP2*** | C | G | -0.04 | -0.032 | -0.003 |
| rs1738475 | 1 | 23409478 | ***HTR1D*** | C | G | 0.022 | 0.056 | -0.034 |
| rs4601530 | 1 | 24916698 | ***CLIC4*** | T | C | -0.02 | 0.004 | -0.028 |
| rs7532866 | 1 | 26614131 | ***LIN28*** | A | G | 0.022 | -0.033 | 0.055 |
| rs2154319 | 1 | 41518357 | ***SCMH1*** | T | C | -0.03 | -0.029 | -0.005 |
| rs17391694 | 1 | 78396214 | ***GIPC2*** | T | C | 0.04 | 0.016 | 0.024 |
| rs6699417 | 1 | 88896031 | ***PKN2*** | T | C | 0.022 | 0.004 | 0.018 |
| rs10874746 | 1 | 93096559 | ***RPL5*** | T | C | -0.02 | 0.053 | -0.075 |
| rs9428104 | 1 | 118657110 | ***SPAG17*** | A | G | -0.04 | -0.025 | -0.013 |
| rs11205277 | 1 | 148159496 | **SF3B4** | A | G | -0.05 | 0.014 | -0.059 |
| rs17346452 | 1 | 170319910 | ***DNM3*** | T | C | -0.04 | -0.012 | -0.026 |
| rs1325598 | 1 | 175058872 | ***PAPPA2*** | A | G | -0.03 | -0.035 | 0.009 |
| rs1046934 | 1 | 182290152 | ***TSEN15*** | A | C | -0.05 | 0.027 | -0.073 |
| rs10863936 | 1 | 210304421 | ***DTL*** | A | G | -0.02 | -0.048 | 0.026 |
| rs6684205 | 1 | 216676325 | ***TGFB2*** | A | G | -0.03 | 0.020 | -0.053 |
| rs11118346 | 1 | 217810342 | ***LYPLAL1*** | T | C | -0.03 | 0.024 | -0.050 |
| rs10799445 | 1 | 225978506 | ***JMJD4*** | A | C | 0.031 | -0.052 | 0.083 |
| rs4665736 | 2 | 25041103 | ***DNAJC27*** | T | C | 0.034 | 0.045 | -0.011 |
| rs6714546 | 2 | 33214929 | ***LTBP1*** | A | G | -0.03 | 0.018 | -0.043 |
| rs17511102 | 2 | 37814117 | ***CDC42EP3*** | A | T | -0.06 | -0.042 | -0.018 |
| rs2341459 | 2 | 44621706 | ***C2orf34*** | T | C | 0.028 | -0.010 | 0.038 |
| rs12474201 | 2 | 46774789 | ***SOCS5*** | A | G | 0.023 | -0.019 | 0.042 |
| rs3791675 | 2 | 55964813 | ***EFEMP1*** | T | C | -0.05 | -0.006 | -0.044 |
| rs11684404 | 2 | 88705737 | ***EIF2AK3*** | T | C | -0.03 | -0.013 | -0.014 |
| rs7567288 | 2 | 134151294 | ***NCKAP5*** | T | C | -0.03 | -0.036 | 0.005 |
| rs7567851 | 2 | 178392966 | ***PDE11A*** | C | G | 0.041 | 0.048 | -0.007 |
| rs1351164 | 2 | 217980143 | ***TNS1*** | T | C | 0.028 | 0.072 | -0.044 |
| rs12470505 | 2 | 219616613 | ***CCDC108/IHH*** | T | G | 0.048 | -0.026 | 0.074 |
| rs2629046 | 2 | 224755988 | ***SERPINE2*** | T | C | 0.025 | 0.031 | -0.006 |
| rs2580816 | 2 | 232506210 | ***NPPC*** | T | C | -0.04 | -0.201 | 0.160 |
| rs12694997 | 2 | 241911659 | ***SEPT2*** | A | G | -0.03 | 0.020 | -0.047 |
| rs2597513 | 3 | 13530836 | ***HDAC11*** | T | C | -0.04 | 0.019 | -0.058 |
| rs13088462 | 3 | 51046753 | ***DOCK3*** | T | C | -0.05 | -0.122 | 0.068 |
| rs2336725 | 3 | 53093779 | ***RTF1*** | T | C | -0.03 | -0.012 | -0.014 |
| rs9835332 | 3 | 56642722 | ***C3orf63*** | C | G | -0.02 | 0.036 | -0.058 |
| rs17806888 | 3 | 67499012 | ***SUCLG2*** | T | C | 0.04 | 0.049 | -0.009 |
| rs9863706 | 3 | 72520103 | ***RYBP*** | T | C | -0.03 | 0.043 | -0.073 |
| rs6439167 | 3 | 130533446 | ***C3orf47*** | T | C | -0.03 | 0.016 | -0.050 |
| rs9844666 | 3 | 137456906 | ***PCCB*** | A | G | -0.03 | -0.008 | -0.020 |
| rs724016 | 3 | 142588260 | ***ZBTB38*** | A | G | -0.07 | -0.057 | -0.010 |
| rs572169 | 3 | 173648421 | ***GHSR*** | T | C | 0.036 | -0.026 | 0.062 |
| rs720390 | 3 | 187031377 | ***IGF2BP2*** | A | G | 0.031 | -0.005 | 0.036 |
| rs2247341 | 4 | 1671115 | ***SLBP/FGFR3*** | A | G | 0.025 | 0.041 | -0.016 |
| rs6449353 | 4 | 17642586 | ***LCORL*** | T | C | 0.071 | -0.048 | 0.119 |
| rs17081935 | 4 | 57518233 | ***POLR2B*** | T | C | 0.031 | 0.007 | 0.024 |
| rs7697556 | 4 | 73734177 | ***ADAMTS3*** | T | C | 0.022 | 0.017 | 0.005 |
| rs788867 | 4 | 82369030 | ***PRKG2/BMP3*** | T | G | -0.04 | -0.131 | 0.092 |
| rs10010325 | 4 | 106325802 | ***TET2*** | A | C | 0.021 | 0.109 | -0.088 |
| rs7689420 | 4 | 145787802 | ***HHIP*** | T | C | -0.07 | 0.036 | -0.105 |
| rs955748 | 4 | 184452669 | ***WWC2*** | A | G | -0.02 | 0.041 | -0.065 |
| rs1173727 | 5 | 32866278 | ***NPR3*** | T | C | 0.036 | -0.020 | 0.056 |
| rs11958779 | 5 | 55037656 | ***SLC38A9*** | A | G | -0.03 | -0.020 | -0.008 |
| rs10037512 | 5 | 88390431 | ***MEF2C*** | T | C | 0.027 | 0.046 | -0.019 |
| rs13177718 | 5 | 108141243 | ***FER*** | T | C | -0.04 | 0.001 | -0.042 |
| rs1582931 | 5 | 122685098 | ***CEP120*** | A | G | -0.03 | -0.016 | -0.009 |
| rs274546 | 5 | 131727766 | ***SLC22A5*** | A | G | -0.03 | -0.075 | 0.047 |
| rs526896 | 5 | 134384604 | ***PITX1*** | T | G | 0.032 | -0.055 | 0.087 |
| rs4282339 | 5 | 168188818 | ***SLIT3*** | A | G | -0.04 | 0.028 | -0.063 |
| rs12153391 | 5 | 171136043 | ***FBXW11*** | A | C | -0.03 | -0.001 | -0.032 |
| rs889014 | 5 | 172916720 | ***BOD1*** | T | C | -0.03 | -0.027 | -0.002 |
| rs422421 | 5 | 176449932 | ***FGFR4/NSD1*** | T | C | -0.03 | -0.009 | -0.024 |
| rs6879260 | 5 | 179663620 | ***GFPT2*** | T | C | -0.03 | 0.026 | -0.054 |
| rs3812163 | 6 | 7670759 | ***BMP6*** | A | T | -0.04 | 0.005 | -0.042 |
| rs1047014 | 6 | 19949472 | ***ID4*** | T | C | -0.03 | 0.027 | -0.056 |
| rs806794 | 6 | 26308656 | **Histone-cluster** | A | G | 0.053 | -0.061 | 0.114 |
| rs3129109 | 6 | 29192211 | ***OR2J3*** | T | C | -0.03 | 0.008 | -0.034 |
| rs2256183 | 6 | 31488508 | ***MICA*** | A | G | 0.035 | -0.014 | 0.049 |
| rs6457620 | 6 | 32771977 | **HLA-locus** | C | G | -0.02 | -0.004 | -0.020 |
| rs2780226 | 6 | 34307070 | ***HMGA1*** | T | C | -0.08 | -0.026 | -0.053 |
| rs6457821 | 6 | 35510783 | ***PPARD/FANCE*** | A | C | -0.12 | -0.058 | -0.063 |
| rs9472414 | 6 | 45054484 | ***SUPT3H/RUNX2*** | A | T | -0.03 | 0.001 | -0.032 |
| rs9360921 | 6 | 76322362 | ***SENP6*** | T | G | -0.05 | 0.031 | -0.079 |
| rs310405 | 6 | 81857081 | ***FAM46A*** | A | G | 0.03 | -0.022 | 0.052 |
| rs7759938 | 6 | 105485647 | ***LIN28B*** | T | C | -0.04 | 0.022 | -0.064 |
| rs1046943 | 6 | 109890634 | ***ZBTB24*** | A | G | 0.022 | 0.045 | -0.023 |
| rs961764 | 6 | 117628849 | ***VGLL2*** | C | G | -0.02 | 0.031 | -0.054 |
| rs1490384 | 6 | 126892853 | ***C6orf173*** | T | C | 0.037 | -0.010 | 0.047 |
| rs6569648 | 6 | 130390812 | ***L3MBTL3*** | T | C | -0.04 | -0.067 | 0.031 |
| rs7763064 | 6 | 142838982 | ***GPR126*** | A | G | -0.05 | 0.077 | -0.122 |
| rs543650 | 6 | 152152636 | ***ESR1*** | T | G | -0.03 | -0.091 | 0.059 |
| rs9456307 | 6 | 158849430 | ***TULP4*** | A | T | -0.05 | 0.004 | -0.054 |
| rs798489 | 7 | 2768329 | ***GNA12*** | T | C | -0.05 | 0.061 | -0.113 |
| rs4470914 | 7 | 19583047 | ***TWISTNB*** | T | C | 0.033 | -0.059 | 0.092 |
| rs12534093 | 7 | 23469499 | ***IGF2BP3*** | A | T | -0.03 | 0.047 | -0.077 |
| rs1708299 | 7 | 28156471 | ***JAZF1*** | A | G | 0.042 | -0.035 | 0.077 |
| rs6959212 | 7 | 38094851 | ***STARD3NL*** | T | C | -0.02 | -0.035 | 0.012 |
| rs42235 | 7 | 92086012 | ***CDK6*** | T | C | 0.055 | -0.059 | 0.114 |
| rs822552 | 7 | 148281567 | ***PDIA4*** | C | G | -0.03 | -0.001 | -0.029 |
| rs2110001 | 7 | 150147955 | ***TMEM176A*** | C | G | -0.03 | -0.039 | 0.006 |
| rs1013209 | 8 | 24172249 | ***ADAM28*** | T | C | -0.03 | 0.037 | -0.066 |
| rs7460090 | 8 | 57356717 | ***SDR16C5*** | T | C | 0.055 | 0.045 | 0.010 |
| rs6473015 | 8 | 78341040 | ***PEX2*** | A | C | -0.03 | -0.014 | -0.018 |
| rs6470764 | 8 | 130794847 | ***GSDMC*** | T | C | -0.05 | 0.056 | -0.103 |
| rs12680655 | 8 | 135706519 | ***ZFAT*** | C | G | 0.03 | 0.032 | -0.002 |
| rs7864648 | 9 | 16358732 | ***BNC2*** | T | G | 0.025 | 0.036 | -0.011 |
| rs11144688 | 9 | 77732106 | ***PCSK5*** | A | G | -0.06 | 0.029 | -0.084 |
| rs7853377 | 9 | 85742025 | ***C9orf64*** | A | G | -0.03 | 0.126 | -0.152 |
| rs8181166 | 9 | 88306448 | ***ZCCHC6*** | C | G | 0.025 | 0.005 | 0.020 |
| rs2778031 | 9 | 90025546 | ***SPIN1*** | T | C | 0.027 | 0.033 | -0.006 |
| rs9969804 | 9 | 94468941 | ***IPPK*** | A | C | 0.028 | 0.022 | 0.006 |
| rs1257763 | 9 | 95933766 | ***PTPDC1*** | A | G | 0.069 | 0.069 | 0.000 |
| rs473902 | 9 | 97296056 | ***PTCH1/FANCC*** | T | G | 0.074 | 0.005 | 0.069 |
| rs7027110 | 9 | 108638867 | ***ZNF462*** | A | G | 0.034 | 0.015 | 0.019 |
| rs1468758 | 9 | 112846903 | ***LPAR1*** | T | C | -0.03 | 0.001 | -0.027 |
| rs751543 | 9 | 118162163 | ***PAPPA*** | T | C | 0.029 | -0.012 | 0.041 |
| rs7466269 | 9 | 132453905 | ***FUBP3*** | A | G | 0.036 | 0.014 | 0.022 |
| rs7849585 | 9 | 138251691 | ***QSOX2*** | T | G | 0.032 | -0.008 | 0.040 |
| rs7909670 | 10 | 12958770 | ***CCDC3*** | T | C | -0.02 | 0.059 | -0.081 |
| rs2145998 | 10 | 80791702 | ***PPIF*** | A | T | -0.03 | 0.024 | -0.049 |
| rs11599750 | 10 | 101795432 | ***CPN1*** | T | C | -0.02 | 0.057 | -0.080 |
| rs2237886 | 11 | 2767307 | ***KCNQ1*** | T | C | 0.043 | -0.036 | 0.079 |
| rs7926971 | 11 | 12654616 | ***TEAD1*** | A | G | -0.02 | -0.041 | 0.017 |
| rs1330 | 11 | 17272605 | ***NUCB2*** | T | C | 0.024 | -0.010 | 0.034 |
| rs10838801 | 11 | 48054856 | ***PTPRJ/SLC39A13*** | A | G | -0.03 | 0.052 | -0.083 |
| rs1814175 | 11 | 49515748 | ***FOLH1*** | T | C | 0.023 | -0.029 | 0.052 |
| rs5017948 | 11 | 51270794 | ***OR4A5*** | A | T | 0.027 | 0.056 | -0.029 |
| rs3782089 | 11 | 65093395 | ***SSSCA1*** | T | C | -0.06 | -0.018 | -0.040 |
| rs7112925 | 11 | 66582736 | ***RHOD*** | T | C | -0.02 | 0.067 | -0.090 |
| rs634552 | 11 | 74959700 | ***SERPINH1*** | T | G | 0.041 | -0.011 | 0.052 |
| rs494459 | 11 | 118079885 | ***TREH*** | T | C | 0.021 | 0.039 | -0.018 |
| rs654723 | 11 | 128091365 | ***FLI1*** | A | C | 0.024 | 0.006 | 0.018 |
| rs2856321 | 12 | 11747040 | ***ETV6*** | A | G | -0.03 | 0.049 | -0.079 |
| rs10770705 | 12 | 20748734 | ***SLCO1C1*** | A | C | 0.031 | 0.013 | 0.018 |
| rs2638953 | 12 | 28425682 | ***CCDC91*** | C | G | 0.036 | 0.119 | -0.083 |
| rs2066807 | 12 | 55026949 | ***STAT2*** | C | G | -0.05 | 0.034 | -0.086 |
| rs1351394 | 12 | 64638093 | ***HMGA2*** | T | C | 0.054 | -0.066 | 0.120 |
| rs10748128 | 12 | 68113925 | ***FRS2*** | T | G | 0.035 | -0.014 | 0.049 |
| rs11107116 | 12 | 92502635 | ***SOCS2*** | T | G | 0.052 | -0.023 | 0.075 |
| rs7971536 | 12 | 100897919 | ***CCDC53/GNPTAB*** | A | T | -0.03 | 0.002 | -0.027 |
| rs11830103 | 12 | 122389499 | ***SBNO1*** | A | G | -0.04 | 0.037 | -0.072 |
| rs7332115 | 13 | 32045548 | ***PDS5B/BRCA2*** | T | G | -0.03 | -0.033 | 0.008 |
| rs3118905 | 13 | 50003335 | ***DLEU7*** | A | G | -0.05 | 0.011 | -0.063 |
| rs7319045 | 13 | 90822575 | ***GPC5*** | A | G | 0.029 | 0.033 | -0.004 |
| rs1950500 | 14 | 23900690 | ***NFATC4*** | T | C | 0.032 | 0.033 | -0.001 |
| rs2093210 | 14 | 60027032 | ***SIX6*** | T | C | -0.03 | -0.011 | -0.023 |
| rs1570106 | 14 | 67882868 | ***RAD51L1*** | T | C | -0.03 | -0.031 | 0.005 |
| rs862034 | 14 | 74060499 | ***LTBP2*** | A | G | -0.02 | 0.022 | -0.045 |
| rs7155279 | 14 | 91555634 | ***TRIP11*** | T | G | -0.03 | -0.008 | -0.021 |
| rs16964211 | 15 | 49317787 | ***CYP19A1*** | A | G | -0.05 | -0.035 | -0.016 |
| rs7178424 | 15 | 60167551 | ***C2CD4A*** | T | C | -0.02 | -0.026 | 0.002 |
| rs10152591 | 15 | 67835211 | ***TLE3*** | A | C | 0.045 | 0.045 | 0.000 |
| rs12902421 | 15 | 69948457 | ***MYO9A*** | T | C | -0.07 | 0.010 | -0.079 |
| rs5742915 | 15 | 72123686 | ***PML*** | T | C | -0.03 | 0.045 | -0.076 |
| rs11259936 | 15 | 82371586 | ***ADAMTSL3*** | A | C | -0.04 | 0.030 | -0.072 |
| rs16942341 | 15 | 87189909 | ***ACAN*** | T | C | -0.13 | -0.062 | -0.072 |
| rs2871865 | 15 | 97012419 | ***IGF1R*** | C | G | 0.054 | -0.053 | 0.107 |
| rs4965598 | 15 | 98577137 | ***ADAMTS17*** | T | C | -0.04 | 0.012 | -0.047 |
| rs11648796 | 16 | 732191 | ***NARFL*** | A | G | -0.03 | -0.013 | -0.018 |
| rs26868 | 16 | 2189377 | ***CASKIN1*** | A | T | 0.03 | -0.068 | 0.098 |
| rs1659127 | 16 | 14295806 | ***MKL2*** | A | G | 0.024 | 0.086 | -0.062 |
| rs8052560 | 16 | 87304743 | ***CTU2/GALNS*** | A | C | 0.039 | -0.019 | 0.058 |
| rs4640244 | 17 | 21224816 | ***KCNJ12*** | A | G | 0.028 | 0.023 | 0.005 |
| rs3110496 | 17 | 24941897 | ***ANKRD13B*** | A | G | -0.02 | -0.011 | -0.012 |
| rs3764419 | 17 | 26188149 | ***ATAD5/RNF135*** | A | C | -0.04 | -0.032 | -0.005 |
| rs17780086 | 17 | 27367395 | ***LRRC37B*** | A | G | 0.035 | -0.003 | 0.038 |
| rs1043515 | 17 | 34175722 | ***PIP4K2B*** | A | G | -0.02 | 0.001 | -0.023 |
| rs4986172 | 17 | 40571807 | ***ACBD4*** | T | C | -0.03 | -0.026 | -0.002 |
| rs2072153 | 17 | 44745013 | ***ZNF652*** | C | G | 0.026 | 0.010 | 0.016 |
| rs4605213 | 17 | 46599746 | ***NME2*** | C | G | 0.023 | -0.027 | 0.050 |
| rs227724 | 17 | 52133816 | ***NOG*** | A | T | -0.03 | -0.069 | 0.042 |
| rs2079795 | 17 | 56851431 | ***TBX2*** | T | C | 0.04 | -0.020 | 0.060 |
| rs2665838 | 17 | 59320197 | ***CSH1/GH1*** | C | G | -0.04 | -0.009 | -0.028 |
| rs11867479 | 17 | 65601802 | ***KCNJ16/KCNJ2*** | T | C | 0.024 | 0.014 | 0.010 |
| rs4800452 | 18 | 18981609 | ***CABLES1*** | T | C | 0.048 | -0.051 | 0.099 |
| rs9967417 | 18 | 45213498 | ***DYM*** | C | G | -0.04 | 0.001 | -0.039 |
| rs17782313 | 18 | 56002077 | ***MC4R*** | T | C | -0.03 | 0.024 | -0.049 |
| rs12982744 | 19 | 2128193 | ***DOT1L*** | C | G | -0.03 | 0.029 | -0.062 |
| rs7507204 | 19 | 3379834 | ***NFIC*** | C | G | 0.028 | -0.059 | 0.087 |
| rs891088 | 19 | 7135762 | ***INSR*** | A | G | -0.03 | -0.075 | 0.050 |
| rs4072910 | 19 | 8550031 | ***ADAMTS10*** | C | G | -0.03 | 0.028 | -0.057 |
| rs2279008 | 19 | 17144303 | ***MYO9B*** | T | C | 0.031 | 0.083 | -0.052 |
| rs17318596 | 19 | 46628935 | ***ATP5SL*** | A | G | 0.029 | -0.022 | 0.051 |
| rs1741344 | 20 | 4049800 | ***SMOX*** | T | C | -0.03 | 0.039 | -0.065 |
| rs2145272 | 20 | 6574218 | ***BMP2*** | A | G | -0.04 | 0.004 | -0.043 |
| rs7274811 | 20 | 31796842 | ***ZNF341*** | T | G | -0.04 | 0.014 | -0.054 |
| rs143384 | 20 | 33489170 | ***GDF5*** | A | G | -0.06 | -0.054 | -0.010 |
| rs237743 | 20 | 47336426 | ***ZNFX1*** | A | G | 0.034 | -0.001 | 0.035 |
| rs2834442 | 21 | 34612656 | ***KCNE2*** | A | T | 0.027 | -0.003 | 0.030 |
| rs4821083 | 22 | 31386341 | ***SYN3*** | T | C | 0.033 | -0.038 | 0.071 |
|  |  |  |  |  | Mean | -0.007 | 0.001 | -0.007 |
|  |  |  |  | Standard deviation | | 0.038 | 0.038 | 0.044 |

Notes: Columns 7 and 8 are plotted in figure 1
